# "The Hospital" Medical Book Supplement.—No. XXI

**Published:** 1909-09-18

**Authors:** 


### "THE HOSPITAL" MEDICAL BOOK SUPPLEMENT.—No. XXI.

CONTENTS.

**Medicine—**
A System of Medicine ... ... ... ... ... ... ... 647
**Surgery—**
A System of Operative Surgery ... ... ... ... ... 648
Sprains and Allied Injuries of Joints ... ... ... ... 648
Synoptic Chart of Cardiac Examination ... ... ... ... 648
**Pathology—**
The Blood in Health and Disease ... ... ... ... ... 649
The Campaign against Microbes ... ... ... ... ... 649
**Hygiene and Public Health—**
The Sanitary Officer's Handbook of Practical Hygiene ... ... 649
The Dietetic Treatment of Diabetes ... ... ... ... ... 649
**Miscellaneous Items—**
The After Treatment of Operations ... ... ... ... ... 650
A Practical Text-book of Midwifery for Nurses ... ... ... 650
One Hundred and Twenty Years of Life and How to Attain Them ... ... ... ... ... ... ... ... ... ... 650
Thoughts and Pastimes ... ... ... ... ... ... ... ... 650
The White Prophet ... ... ... ... ... ... ... ... 650

### MEDICINE.

A System of Medicine. Edited by William Osler, assisted by Thomas McCrae. (Oxford Medical Publications. Vol. VI. Pp. 800. Price 30s. net.)

The sixth volume of this system reaches a higher level of general excellence than some of its predecessors, and is on the whole extremely good and interesting. The peculiar interest of it lies partly in the fact that it deals with a number of the more mysterious maladies which plague us, maladies about which one feels that at any moment some new experimental undertaking or original generalisation may give us a key to them.

The first part of the book is devoted to diseases of the urinary system. The subject is comprehensively and sufficiently dealt with, and includes an excellent survey of the anomalies of urinary secretion from the pen of Dr. Garrod, whose name in this connection has justly won respect on this side. Nevertheless one concludes the section with an uncomfortable conviction of the truth of the statement made by the assistant editor in his prefatory chapter. "In the last twenty-five years," he says, "there has been a great mass of work done with reference to the problems presented by urinary secretion, and one must admit that there has been no advance made at all commensurate with the energy expended, or at all comparable with the increase of knowledge concerning other apparently less important organs." Yet the contributors have done their work well, and the reader will find here a good account of the present position of our knowledge in this department.

Part II., dealing with the diseases of the ductless glands, is entirely from the pen of Dr. George Dock. If this gentleman is fortunate in his subject—which as we have hinted has a peculiar attraction of its own—he is none the less to be congratulated upon his method of presenting it. He is a clear and cogent writer, and has obviously spent much painstaking research in the preparation of his material. His articles are excellent examples of compression, effected without the sacrifice of intelligibility; and for this he may be sure that reveiwer and student will be equally thankful. We have had occasion, in reviewing preceding volumes of this series, to carp at the diffuseness and verbosity of several contributors. It is but just, therefore, that we should note the relative freedom of this volume from these defects; and particularly the fine example set to his colleagues by Dr. Dock.

The third part is concerned with diseases of "obscure causation." It is somewhat surprising to find under this heading only five diseases, and still more surprising to find Hodgkin's disease, Arthritis deformans, and Osteomalakia cheek by jowl with Astasia abasia and Adiposis dolorosa. We must suppose that some plan underlies this arrangement, but confess our inability to fathom it. The chapter on Lymphadenoma is well enough in its way, but unsatisfying. It is the custom of some writers for large systems of medicine to be content with accumulating, at a most meritorious expenditure of pains, the opinions and data of previous workers in a given field. But we wish to enter an emphatic protest against assuming that a writer who has done so much has done all that is required of him. It is to be presumed that when a man is invited to contribute an article on a special disease he is so invited because he has the reputation of a specialist in that disease. At all events, by the time a man of ordinary ability has devoted to a subject study enough to produce such an article as this on Hodgkin's disease, obviously the result of laborious compilation, he must have come to some definite, or more or less definite, conclusions of his own as to the nature of the malady. Yet we are not given them. Surely authoritative writers, after a comprehensive study of the data before them, should summarise the evidence for the benefit of their readers, and say, "having weighed all the evidence to which I have had access, I am of opinion that (to take the case in point) Hodgkin's disease is an infective granuloma." Or the reverse, as the case may be. As it is, the reviewer, who lays no claim to be a specialist in Hodgkin's disease, leaves the business throughly puzzled as to which of many contradictory views carries the greatest conviction to a man who has made an elaborate study of the subject.

Dr. McCrae has dealt as clearly as the murkiness of the subject permits with the variety of diseases commonly comprised by the name Arthritis Deformans. In the next, the fifth, part are three articles on vaso-motor and trophic disorders from the able pen of Prof. Osler. It is of course idle to repine that we cannot have an Osler to write all the articles, but if writers only learnt to appreciate the added conviction lent to any essay on a medical subject by the inclusion of personal experience and opinion, they would write, as he does, in the first person, and tell us always what they think about the business in hand.

The final section deals with the medical aspects of life assurance. The statements in it are based upon the experience of American offices. As is usual with text-book pronouncements on this subject a degree of thoroughness is insisted upon which, though often urged in this country, is probably never acted upon even by those who urge it. The truth is that there is so large an element of business and competition in life assurance that what might be best from the purely medical point of view has often to give way to considerations of simplicity and freedom from vexatious delays. Whatever medical examiners may say, their prime function is to obviate a selection against the office they serve. They are there to exclude the man who, feeling that ill-health is creeping upon him, is in haste to insure himself. Given safety from this danger, all that an office has to do is to increase its business to such an extent as to allow fair play to the law of averages upon which its premiums are based.

### SURGERY.

A System of Operative Surgery. By various authors. Edited by F. F. Burghard, M.S.Lond., F.R.C.S.Eng. (London : Henry Frowde, Oxford University Press, and Hodder and Stoughton, Warwick Square, E.C. 1909. Vol. IV. Pp. xxvi + 687; illustrations 351. Price 36s.)

The fourth volume of Mr. Burghard's system of operative surgery consists of a series of articles upon those departments of surgery which are essentially the domain of specialists. Mr. Bland-Sutton is the only general surgeon who contributes to this volume; but he writes on a subject which he has made peculiarly his own—namely, abdominal gynæcological operations—and his present contribution is worthy of his reputation. He writes with an enviable lucidity, and this is particularly the case of his description of a radical abdominal hysterectomy, an operation which is associated in this country with the name of Wertheim. We see no advantage in the use of the term "metastatic bacteriæmia" instead of puerperal sepsis. It is perhaps more scientific, but it is not as expressive as the older name.

Dr. Phillips' article upon vaginal operations is also extremely good, and we have nothing but admiration for his description of operations for the repair of complete lacerations of the perineum. It is both terse and helpful, and the illustrations which accompany the text give one an extremely clear idea of the procedure. Mr. Mayou writes upon operations upon the eye. The arrangement is good, and his descriptions are clear. The nomenclature is up-to-date and the illustrations are excellent. The operations upon the ear have been entrusted to Mr. Hunter Tod, who is already well known as a writer on this particular subject. We are most favourably impressed by his treatment of operations upon the mastoid process, a difficult thing to describe succinctly. But Mr. Tod succeeds entirely, especially in pointing out the essential differences between Schwartze's operation and the more radical one known as the Küster-Bergmann or Schwartze-Stacke operation. He also gives a most interesting account of the history of mastoid operations. His description of skin-grafting after mastoid operations is as lucid as the rest of his work. A very adequate account is also given of the operations which may be necessitated by the extension of septic processes from the ear—*e.g.* intracranial abscess and thrombosis of the jugular vein.

Laryngeal operations are described by Mr. Harmer, of St. Bartholomew's Hospital. One is struck on reading his article by the modifications in the technique of this science which have resulted from the perfecting of the direct method of laryngoscopy. A most instructive chapter is one in which the results obtained by the different extra-laryngeal operations are compared; the value of laryngotomy, as a preliminary manœuvre in operations upon the upper air passages is insisted upon, a contention in which all practical surgeons, who have any experience in these operations, must agree. In a chapter dealing with intubation of the larynx, Mr. Harmer writes: "Although intubation has received extensive trial, the published results show great variations and do not prove that intubation is superior to tracheotomy, but rather the reverse." This is a verdict which commands our entire concurrence. The operations on the nose are admirably dealt with by Professor St. Clair Thompson. The descriptions of operations for septal deformities and of those upon the frontal sinus are particularly excellent. We have the fullest confidence in recommending this volume to the profession. The articles are characterised throughout by a singular lucidity and freedom from unnecessary verbiage, which is as welcome as it is unusual.

Sprains and Allied Injuries of Joints. By R. H. A. Whitelocke, M.D. (Edin.), F.R.C.S.; Lichfield Assistant Pathologist, Edinburgh Royal Infirmary. (London : John Bale, Sons and Danielsson. 2s. 6d. net.)

In the treatment of many injuries of bones and joints the medical profession as a whole has in the past been compelled to readjust its conceptions of what is and what is not correct and proper. A generation ago the acuteness of Mr. Wharton Hood perceived that in one or two respects the bonesetter acted on principles which were surgically sounder and gave better results than those current among the profession, and his success compelled thoughtful surgeons to recognise that the enforcement of prolonged rest as a fundamental part of the treatment of all injuries is in many cases a mistake. This error, partly due to an overstraining of the ideas propounded in Hilton's classical work on rest and pain, is even now not wholly abandoned; but it is hardly to be imagined that it will survive much longer the onslaught of such uncompromising opponents as Mr. Whitelocke. As the author points out, the extension of *x*-ray examination to all kinds of obscure articular injuries has revealed all kinds of hitherto unsuspected fractures and fracture-dislocations, and the Workmen's Compensation Acts of recent years have made diagnosis, treatment, and prognosis of all injuries more than ever of importance. In a series of highly practical and well illustrated chapters, the author considers sprains in general and then their immediate and remote sequelæ, passing on subsequently to individual articulations and the especial difficulties and complications there encountered. Internal derangements of the knee very justly receive a separate chapter; and injuries to muscles and tendons, with an account of the indications for and against massage, movements, and exercises complete the volume. Throughout the book the principles of treatment in which Mr. Whitelocke believes, and which he has the most ample opportunities of practising and testing as a surgeon in a University town, are carefully laid down and explained. Nor is there anything of the blind partisan about his attitude: open operations are freely advocated for various conditions, but only on definite indications, and never if there is any equally useful alternative. So with massage, exercises, and ambulatory treatment of injuries generally. Mr. Whitelocke's surgery is in fact eminently sane, and his monograph can be recommended to all as a helpful contribution to this subject; to those who have to attend any section of the athletic world in which injuries are frequent, it will prove indispensable.

---

Synoptic Chart of Cardiac Examination. Arranged by John D. Comrie, M.A., B.Sc., M.B., F.R.C.P.E., Assistant Pathologist, lately Clinical Tutor, Royal Infirmary of Edinburgh. (London : John Bale, Sons and Danielsson, Limited. 1909. 2s. 6d. net.)

The author of this Chart has produced what we should describe as an ingenious toy, but we are afraid that it is not likely to prove of practical value to medical students. The Chart consists of an outline figure of a man, with certain apertures in it, behind which, by means of a moving tape, the names of different heart lesions are made to appear synchronously with the chief abnormal physical signs to which each lesion in its turn gives rise. The Chart is accompanied by a twelve-page pamphlet, in which are summarised the numerous symptoms and physical signs of various heart diseases. We think the Chart may be of interest to those who already know all that it can teach, but we doubt whether those who do not know will be likely to learn much from it.

### PATHOLOGY.

The Blood in Health and Disease. By R. J. M. Buchanan, M.D., F.R.C.P., Professor of Forensic Medicine in the University of Liverpool, etc. Pp. xvi. + 318, with numerous illustrations. (London: Henry Frowde, Hodder and Stoughton. 1909. Price 12s. 6d. net.)

This volume is another of the "Oxford Medical Publication" series, and attains a high standard in the matter of printing and illustration. Some of the coloured diagrams of blood corpuscles in it are amongst the best we know. After dealing with the methods of estimating blood corpuscles and hæmoglobin, and after describing in detail the various processes for fixing and staining blood films, the author devotes the rest of the book to the changes the blood undergoes, not only in the more definite blood diseases, but also in the majority of other conditions in which there are any blood changes at all. Nevertheless, we feel that there are a good many points that one would expect to find in the book, and which have yet been omitted. We have scarcely room to refer to all of these, and we must be content with mentioning one or two only. In a work to which, according to the title, one should be able to refer for any points in regard to the blood upon which one needed information, one is surprised to find Widal's clumping reaction is but vaguely described, and that the dilution of 1 in 200 is not insisted upon as it ought to be. Malaria, trypanosomiasis, and other parasitic affections of the blood have been entirely omitted upon the ground that they belong to the special dominion of tropical diseases. Although Dr. Buchanan's book is good so far as it goes, practitioners may be disappointed by failing to find in it many things which they might have expected to from its title.

The Campaign Against Microbes. By Etienne Burnet, M.D., of the Pasteur Institute, Paris. Translated by E. E. Austen, F.Z.S. (London: John Bale, Sons and Danielsson, Limited. 1909. Price 5s. net.)

The title of this book gives little idea of its subject-matter. This is no treatise upon disinfectants, but a series of articles upon Cancer, Tuberculosis, Tetanus, Sleeping Sickness, Enteritis and Intestinal Microbes, and Variola. Each article is drawn up upon similar lines, the author tracing in a clear and concise way the chief experiments and observations that have led up to the present stage of knowledge upon each of the above subjects. The evidence is given fairly and without bias; even though Dr. Burnet obviously holds the view that a microbe or a parasite is at the root of cancer, he gives the pros and cons with such honesty that he clearly wishes the reader to draw conclusions from the evidence rather than from his own personal opinion. Those who are already familiar with the experiments discussed may find too little detail in the volume; but practitioners who would like to be made *au fait* with the lines along which laboratory workers are attacking the problems of the campaign against these diseases will find this book most pleasant and useful reading. It is likely to be read by certain of the laity also, and with advantage in many ways, particularly as a summary of some of the immense benefits that are being derived from physiological, bacteriological, and other experimental methods of research. It is good to see the esteem in which Dr. Jenner is held in France. The account of him in the article on Variola is most pleasing. It is also interesting to note that it is not in England only that people accuse themselves of being all behind the times. Dr. Burnet repeatedly laments that France moves too slowly in matters of public health.

### HYGIENE AND PUBLIC HEALTH.

The Sanitary Officer's Handbook of Practical Hygiene. By C. F. Wanhill, Major R.A.M.C., M.R.C.S.Eng., L.R.C.P.Lond., D.P.H.Eng., and W. W. Beveridge, D.S.O., Major R.A.M.C., M.B., C.M.Ed., D.P.H.Camb. (London: Edward Arnold. 5s. net.)

This book has been written to cover the needs of the military branches of the medical profession, and the general scheme is that on which the training in the Hygiene Laboratories, Royal Army Medical College, is carried out, and there found to be satisfactory both for military purposes and for preparation for the examinations for the diploma of public health. The authors, who are respectively assistant professor of hygiene, Royal Army Medical College, and analyst to the Army Medical Advisory Board, state in the preface that processes and apparatus not usually found in small laboratories in distant lands are as useless as a cookery book to an inhabitant of the Soudan. They also state that teaching of the subject is not attempted, it being assumed that the reader has had previous training and requires merely to refresh his memory with regard to details. The book contains chapters on Water, Sewage, Ventilation, Analysis of Foods, Analysis of Beverages, Calculation of Diets, and Bacteriology, together with two appendices dealing respectively with chemical and bacteriological details. It has a well-arranged index, and is interleaved throughout with blank pages for additional notes. Very clear instructions are given as to the best methods of water and sewage analysis, including the preparation of standard solutions. Useful notes on the various processes, together with examples, have been given. In the instructions given for estimating the amount of carbon dioxide in air by Pettenkoffer's process we should prefer to advise the student to dry very carefully the bottle before taking the sample of air into it. The water which is left in the bottle after filling with water and inverting the same will produce variable results in the amount of carbon dioxide estimated. We prefer the use of bellows in taking samples of air. The chapters on the Analysis of Foods and Beverages are clearly arranged. In the chapter on Bacteriology a table indicating the cultural differences between various bacilli is a useful addition. Details as to the preparations and standardisation of media have been given. We are sure that the book will be of the utmost value to those students who have attended the authors' classes; but as a rule students who have attended classes in preparation for the examinations for the diploma of public health elsewhere prefer to rely on the practical notes given to them by their own teachers, supplemented by one of the larger text-books in common use.

The Dietetic Treatment of Diabetes. By B. D. Basu, Major I.M.S. (retired). (The Panini Office, Bhuvaneshvari, Ashram, Allahabad, 1909. Rs. 1-8-0.)

An excellent little work, which should prove of decided value to every practitioner. It is written in a thoroughly practical fashion, and is somewhat more than a mere compilation of extracts from the authorities. What it gives is up to date and lucidly stated. Major Basu's little work ought to become a popular one; at any rate we can thoroughly recommend it to the practitioner who is interested in the subject with which it deals.

### MISCELLANEOUS ITEMS.

The After Treatment of Operations. By P. Lockhart Mummery, M.B., F.R.C.S. (London: Baillière, Tindall, and Cox. Third edition. 1909. Price 5s. net.)

When this well-known text-book was first published, six years ago, the subject with which it deals was almost entirely neglected in surgical literature; even in the largest and most pretentious works on operative surgery it received very inadequate attention. In consequence, dealing as it does with an aspect of surgery very important indeed to the general practitioner and to the surgeon practising in the country or abroad, it proved a perfect godsend to very many medical men and met with a well-deserved success. Since then much more attention has been paid to the management of patients after operation, and the progress made in this field has been very great. The author himself observes in the preface to this new edition that probably in no branch of surgical technique has improvement been more marked, and it is not difficult to agree with him. He has, in revising the chapters for republication, chosen with discretion those which have gained most universal approbation of these changes and improvements; to have included every suggestion would no doubt have resulted in a cyclopædia instead of a compact and convenient manual. Still, one misses one or two of the newer points which might, perhaps, have been included with advantage. The index remains, as before, much too fragmentary to be of any great use; but, apart from these minor criticisms, Mr. Mummery's book well maintains its old reputation as one of those inexpensive monographs which, to the house-surgeon, the practitioner, and all those who may be called upon to undertake the care of an operation case without a ripe experience, is worth far more than a shelf full of bulkier and dearer, but less practical, text-books.

A Practical Text-book of Midwifery for Nurses. By Robert Jardine, M.D. Edin., M.R.C.S. Eng., F.F.P. and S. Glasg., F.R.S. Edin. With 49 illustrations. Fourth edition. Pp. 304 + xv. (London: Henry Kimpton. 1909. Price 5s.)

Careful perusal of the fourth edition of this little book fully explains the reason of its continued popularity. Written in simple language, with clear definitions and accurate statements of fact, it would seem to fulfil all the reasonable requirements of the class of student for which it is primarily intended. One or two small defects are, however, noticeable, the most serious of which is the absence of a definition of the "lie" of the fœtus. The volume is well printed, in clear type, and the illustrations are excellent. It can be recommended to nurses, and will repay perusal by medical students who are in want of a concise account of their duties on the "district."

One Hundred and Twenty Years of Life and How to Attain Them. By Charles Reinhardt, M.D. Pp. 50. (The London Publicity Company, Limited. 1909 Price 1s.)

There are a number of publications now appearing upon the general subject, "Lactic Ferments, the cure for nearly everything," and this is one of them. We quite believe that there is a great deal in the Bulgarian bacillus treatment; but we think that more weight is being put on it than it will carry. The arguments seem to be based upon two separate ideas, and we are not convinced that either of them is correct. The first argument is as follows: In proportion to our population we have only one centenarian in this country to one hundred and eighty-seven in Bulgaria. In the latter country sour milk has been the national diet for ages; therefore if only we drank sour milk too we should be centenarians also. The second argument is as follows: Bulgarian lactic acid bacilli, introduced into the bowel, inhibit the growth of other putrefactive organisms there; intestinal putrefaction causes auto-intoxication and premature senility; therefore lactic bacillary treatment leads to longevity. We think that the conclusion is unwarranted in the first argument, and the second premiss has never been proved in the other. We do not believe that the sour milk treatment is the panacea Dr. Reinhardt and other authors would make it out to be. The booklet has no index. It mentions several firms from which the various articles and products described in the text are to be obtained, and it concludes with a series of advertisements of those firms themselves. Two points need special mention. We think the Cyllin treatment as a preliminary to the use of the lactic ferment is likely to be good; and the practical suggestion that "typhoid carriers" may be curable by the lactic acid bacillus (p. 27) opens up a means of relief to a condition which has recently become one of considerable importance and difficulty.

Thoughts and Pastimes. By M. E. R. (London: Kegan Paul, Trench, Trübner and Co. 1909. Price 3s. 6d. net.)

This volume of verse is published with a view to aiding the funds of the Great Ormond Street Hospital for Sick Children, and it is dedicated by permission to Her Royal Highness the Princess Royal, vice-patron of that institution. We approve highly the motive and intentions which have inspired the publication of the volume, but regret we cannot honestly find much else in it to approve. The verses are crude in the extreme: the piece entitled "The British Empire" is enough to make angels, much more reviewers, weep; and there are other pieces nearly as bad.

The White Prophet. By Hall Caine. (London: Wm. Heinemann, 1909. Two vols., small 8vo. 4s. net.)

This book is noticeable as the first of Mr. Heinemann's new library of modern fiction series. As such it is undoubtedly interesting, regarded merely from a technical point of view. The type is long primer leaded, and the spacing is everywhere excellent. The paper is unglazed with wide margins, and there is a really admirable scarcity of printers' errors in the text. Add to this that each volume is light in weight and convenient in size, and it will be seen that the medical critic, who should, in discussing the make-up of a book, pay due regard to the fact that there are such things as eye strain and visual errors—a fact which the majority of publishers utterly disregard—can have only praise for Mr. Heinemann's venture. The series should be a popular one with book-lovers—if the literary fare provided is as good as the typographical medium in which it is served. Unfortunately the series opens with a weak story which has neither much interest nor literary merit, and which will not add greatly to Mr. Caine's reputation.

### BOOKS RECEIVED.

The Panini Office, Bhuvaneshvari Ashram, India.

"The Dietetic Treatment of Diabetes." By B. D. Basu.

